# Point-of-care ultrasound in resource-limited settings: the PURLS fellowship

**DOI:** 10.1186/s13089-020-00159-6

**Published:** 2020-03-20

**Authors:** Samuel L. Burleson, David C. Pigott, John P. Gullett, Christopher Greene, Courtney B. Gibson, Scott Irvine, Daniel Kaminstein

**Affiliations:** 1 Department of Emergency Medicine, University of Alabama at Birmingham Hospital, Old Hillman Building Suite 251, 619 19th St S, Birmingham, AL 35249 USA; 2 Department of Emergency Medicine, Augusta University, Augusta, GA USA

**Keywords:** Point-of-care ultrasound, Tropical infectious disease, Resource-limited settings, Fellowship

## Abstract

**Background:**

The role of point-of-care ultrasonography (POCUS) is rapidly expanding in both resource-rich and resource-limited settings (RLS). One limitation to this rapid expansion has been the lack of educators adequately trained to teach this user-dependent skill. This is particularly true in RLS, where disease presentations, infrastructure limitations, and approach to medical education present unique challenges to the direct application of resource-rich emergency department POCUS curricula.

**Objectives:**

We describe the point-of-care ultrasound in resource-limited settings (PURLS) fellowship, a novel curriculum designed to provide advanced training and expertise in clinical care and POCUS application and education in RLS.

**Conclusion:**

Our curriculum design is one approach to create context-specific POCUS education for use in RLS, thereby improving patient care.

## Introduction

Improvements in portability, image quality, and ease-of-use are driving the expansion of point-of-care ultrasound (POCUS) for screening, diagnosis, and procedural guidance [1]. Emergency clinicians employ a wide variety of POCUS applications to make rapid diagnoses, guide resuscitation, and improve procedural success and decrease complications [2, 3].

As POCUS use expands, accreditation bodies in the United States (USA) [4] and internationally [5] are requiring or recommending POCUS incorporation into emergency medicine training programs. In the USA, this training is primarily provided by emergency ultrasound (EUS) fellowship-trained faculty. The goal of fellowship training is to create leaders in POCUS education [6]. As a result of the increased use of POCUS there has been a proliferation of USA EUS fellowships, with 115 active programs at the time of this writing [7]. In addition, POCUS fellowships are developing in other specialties such as Internal Medicine, Pediatrics, and Critical Care. Few, if any, such training opportunities exist for clinicians living and working in resource-limited settings (RLS), or for those planning to.

The goal of the fellowship outlined below is to equip graduates to appropriately use and teach contextualized ultrasound skills in RLS. The benefits of POCUS in RLS are increasingly recognized. Maru et al. deem X-ray and ultrasound the “key” imaging modalities primary and emergency care in rural and RLS [8]. Unfortunately, traditional radiology modalities, even when available in RLS, require expensive equipment, maintenance, trained staff, and specialists to interpret images [9], and the costs of such studies or the distance of travel required may be prohibitive. Trained clinicians with POCUS may alleviate some of these limitations [10]. Portable hand-held ultrasound systems can be purchased for $2000 USD, adding a solar charger and external battery brings the total cost for an ultrasound system that does not require a stable power supply to $2200 USD. While this is still expensive for any individual provider, it represents a dramatic drop in cost from the laptop-based systems costing tens of thousands of dollars that were the primary tool for providers in RLS up until a few years ago. Many clinicians with POCUS expertise have brought their skills to RLS, and while there has been a huge benefit of this expertise, there are reasons ultrasound use and training in these settings needs to become more context specific.

Many POCUS applications thought to have the highest utility in the RLS are familiar to developed-world clinicians. POCUS use in peripartum care, trauma, heart failure [11], undifferentiated hypotension or dyspnea [12, 13], and deep venous thrombosis [14] in RLS are all well-described. In settings lacking confirmatory diagnostics, POCUS may be even more valuable to a patient’s clinical evaluation than in a developed setting. POCUS use in RLS may impact the clinical management of 50–70% patients [15, 16].

A growing body of literature documents POCUS use in the management of tropical infectious diseases including extrapulmonary tuberculosis in Human Immunodeficiency Virus (HIV), cystic echinococcosis, liver abscess, schistosomiasis, lymphatic filariasis, visceral leishmaniasis, and viral hemorrhagic fever [17, 18]. These applications are not included in most POCUS curricula and may yield unique ultrasound findings or apply traditional ultrasound exams to new contexts (such as the application of the FAST exam to extrapulmonary tuberculosis or dengue). Belard et al. conclude the potential of POCUS in tropical infectious disease management appears “huge and increasing”, but high-quality training and standardized protocols are the keys to continued growth [17]. Becker and colleagues concur that the lack of POCUS-trained clinicians, specifically qualified instructors and educational opportunities, inhibits the broader implementation of ultrasound in RLS [15].

Many POCUS training strategies in RLS rely initially on external expertise, usually provided by clinicians with extensive POCUS experience requiring years to develop or by EUS fellowship-trained faculty. Unfortunately, resource limitations and tropical diseases create challenges when attempting direct translation of emergency POCUS to these settings. Van Hoving and Lamprecht describe discrepancies between the standardized South African POCUS curriculum (modeled after resource-rich settings) and the actual diseases seen by providers [19]. Salmon and colleagues note that the direct transfer of curricula to RLS “fails to acknowledge the unique challenges, needs, and disease burdens” of those settings [20]. Henwood et al. emphasize the importance of a needs assessment involving stakeholders at multiple levels to determine which applications should be included in any potential curriculum [21]. Burdick et al. note that training in International Emergency Medicine (IEM) or Global Health “needs to emphasize knowledge of both biologic and cultural aspects of care in specific environments” and recommend against applying resource-rich systems directly to RLS without careful analysis of actual effects and limitations [22]. The same could be said of the application of POCUS curricula.

Clinicians and especially educators in RLS therefore require expertise in tropical diseases and POCUS applications not included in USA EUS fellowship curricula. They also must understand regional differences in epidemiology, pathology, and resources. A structured fellowship curriculum can provide this expertise, while also developing the educational capacity to then provide training to other clinicians in RLS in a “train the trainer” model tailored to the needs of that particular setting [9, 11, 20]. We describe the point-of-care ultrasound in RLS (PURLS) fellowship, a novel Emergency Medicine fellowship. Conceptualized to meet the curricular guidelines of USA EUS fellowships [6] while providing clinical and ultrasound experience in RLS and tropical medicine, the curriculum develops fellows into effective clinicians in RLS and leaders in POCUS training in any clinical setting.

## Discussion

### Curriculum description

The PURLS fellowship is an 18-month curriculum integrating clinical faculty practice at an academic ED with clinical experience in a variety of RLS and international training sites. This includes advanced hands-on POCUS experience and teaching in both resource-rich and RLS. An overview of the curriculum is provided in Table 1. Table 2 details the PURLS timeline.

**Table 1:** Overview of PURLS fellowship curriculum components

Content area	Frequency	Length
***Emergency ultrasound***		
Bedside scanning and teaching shifts with students and residents	Weekly	Longitudinal
Advanced scanning skills (cardiac, musculoskeletal, hepatic, OB/pelvic, procedural)	Monthly	Longitudinal
Image review, quality assurance, program administration	Weekly	Longitudinal
***Tropical medicine and global health***		
Diploma in tropical medicine and hygiene		9–12 weeks
Clinical experience in RLS	Variable	8–10 weeks
Global health conference	Every 2 weeks	Longitudinal
Humanitarian response or trip leadership course		2 weeks
***Point-of-care ultrasound in resource-limited settings***		
Clinical ultrasound in tropical infectious diseases course		2 weeks
Clinical and teaching experience in RLS	Variable	Longitudinal while abroad
Research

**Table 2:** Summary of a fellow’s international and domestic work

Timeline	Location	Description of work	Length
July–August	United States	Routine clinical and fellow duties	
September	Tanzania	POCUS scanning and education at an academic tertiary referral hospital	3 weeks
October–November	United States	Routine clinical and fellow duties	
December	The Gambia	Rural primary care clinics with no imaging besides POCUS	2 weeks
January–March	Peru	Clinical ultrasound in tropical infectious diseases course	1 week
		Diploma of tropical medicine and hygiene—POCUS on daily inpatient rounds	9 weeks
April	United States	Humanitarian response course	2 weeks
May–August	United States	Routine clinical and fellow duties	
September	Tanzania	POCUS scanning and education at an academic tertiary referral hospital	2 weeks
October–December	United States	Routine clinical and fellow duties	

Clinical experience in the RLS is fluid by design to adapt to the aspirations of the individual fellow, and includes a combination of hospital work, primary care, and refugee/disaster medicine. Most clinical work will take place at sites or with organizations with established relationships with fellowship faculty, in order to facilitate a more coherent longitudinal experience for the fellow, faculty, local partners, and ongoing projects.

To improve clinical expertise and provide context for the expansion of ultrasound in tropical medicine and RLS, the fellow must complete a clinical tropical medicine course [23–26] and sit for the Diploma of Tropical Medicine and Hygiene (DTM&H) exam. This provides the framework on which fellows build their clinical and POCUS experience and allows fellows to better understand the diseases and health infrastructure in other settings. Fellows also have the opportunity to complete courses in medical trip leadership or humanitarian response [27, 28].

Fellows participate with faculty in global health teaching discussions every 2 weeks, focused on tropical medicine case studies, trip planning, logistics, and refugee health. Time is also spent outlining research goals, and integrating clinical practice and POCUS into local patterns according to needs assessments or published guidelines. This teaching complements the clinical tropical medicine course and provides longitudinal mentorship focused on practice in RLS while the fellow works domestically. Conferences are also an opportunity to address topics not encountered by the fellow in their personal experience.

Fellows integrate POCUS into clinical practice in all settings. They participate in and teach advanced training courses [29] focusing on ultrasound in tropical infectious disease which emphasize the unique role of POCUS in both tropical infections and RLS. Fellows and faculty also train local providers in discrete basic POCUS protocols, such as the FASH or obstetric exams, or POCUS-guided vascular access. These education modules are driven by local needs assessments or by the requests of local partners. Fellows also complete a research project integrating POCUS, education, and practice in the RLS.

When working in the USA, fellows fulfill a traditional EUS fellowship role, including resident and medical student education, image review, QA, EUS program management, research, and advanced training in specific areas as directed by the needs of the fellow. Each aspect is intended to equip the fellow with the skills to implement a fully functioning POCUS training program. These roles allow faculty to evaluate the fellow’s practical competencies and develop the teaching skills of the fellow prior to independent work in the RLS.

Advanced echocardiography training includes scanning sessions in the echocardiography laboratory and echocardiographic interpretation with a board-certified cardiologist. Pathology relevant to RLS is emphasized, such as rheumatic heart disease, pulmonary hypertension (often secondary to chronic parasitic infections such as schistosomiasis), and hypertensive heart disease, all of which are common in these settings [30–32], and often diagnosed in advanced stages [30, 32, 33].

Fellows have the opportunity to scan with expert ultrasound technicians in the resource-rich setting to gain experience with applications not often included in POCUS curricula, but frequently utilized in RLS, such as hepatic and splenic ultrasound [21]. Fellows may also work with an emergency and sports medicine physician in clinic to improve skills in diagnostic musculoskeletal POCUS, regional anesthesia, and ultrasound-guided procedures.

Faculty augment this training with supervised scanning in RLS, case studies, and image review over the course of the fellowship. With new technology, image review can be performed remotely and almost instantaneously, allowing fellows to consult faculty when encountering new pathology in the field. Fellows also participate in an ultrasound training course in RLS run by a faculty member with both expertise in tropical medicine and ultrasound. This provides the fellow an opportunity to participate in training other providers with limited ultrasound skills in a low-resource environment where tropical diseases are common.

### Goals and objectives

The authors believe the combination of clinical experience and teaching in RLS, formal training in tropical infectious diseases, and training in advanced POCUS applications enables fellows to expertly obtain and interpret images, then apply those findings appropriately to the patient and the clinical context. The authors envision the PURLS curriculum producing graduates who are competent to provide excellent contextualized patient care in RLS, employ POCUS to improve the care of individual patients in RLS, and to train other providers to use the modality, both those from the resource-rich setting gaining new skills for global health work and those native to RLS. Focused teaching, research, and experience during the fellowship further equip graduates to develop appropriate curricula that address the assessed needs of patients and clinicians in a particular setting and implement curricula such that POCUS usage can become self-sufficient in regards to local expertise, quality assurance, and supplies.

The PURLS curriculum combines aspects of both EUS and IEM fellowships, inculcating fellows with both advanced ultrasonography skills and the flexibility, cultural competence, and knowledge of disease burdens in RLS. Our curriculum emphasizes the complementary aspects of both fellowships, providing a broader perspective and skill set than either fellowship alone.

### Future changes

These conclusions and the limitations to follow represent the views of one fellow (SLB) and fellowship faculty upon completion of the fellowship. Future changes to the curriculum could include the development of hands-on short-term courses in a variety of settings and specialties to increase dissemination of the content and provide supervised settings for competency development in a greater number of learners. The development of a comprehensive, distributable, interactive, asynchronous educational resource would benefit the fellow and other learners, especially with rarer pathology, or that with limited geographic distribution. Finally, POCUS use in tropical infectious disease is growing rapidly, so new applications will likely need to be added to the curriculum continually.

### Limitations

Limitations for our particular fellowship include the necessity of prior Emergency Medicine residency training, basic POCUS competency, and a broad knowledge base encompassing multiple subspecialties. The 18-month duration of the fellowship is a significant time commitment. The fellowship curriculum could potentially be applied to other specialties, such as internal, family, or critical care medicine, though the fellowship itself might require more time, since emergency medicine graduates have more extensive ultrasound training built into residency in the USA. One dual-boarded Internal Medicine and Pediatric physician has completed an earlier iteration of this fellowship model, so this concept has been trialed outside of Emergency Medicine.

Another limitation is that this fellowship and curriculum is based on model of advanced training that is primarily found in Canada and the United States. Other countries where specialty training and graduate medical education has a different structure will have trouble following the exact format of the fellowship and creating a financially viable model that is similar to what has been done here. We believe the primary concept of focused training that combines ultrasound with tropical medicine and an advanced knowledge of care delivery in RLS is achievable in most training settings, but may require modification of the timeline and cost specifics.

To allow adequate time for international and educational responsibilities, fellows work approximately half the clinical shifts of full-time academic faculty and are paid commensurate with the clinical work completed, which is consistent practice among all Emergency Medicine fellowships at our institution. Financial resources available to fellows for training and travel are also a potential limitation, but at our institution, a portion of the fellow salary from clinical work is withheld to pay for travel and other expenses, an arrangement acceptable to prior fellows.

The need for significant departmental resources, likely including a pre-existing EUS fellowship and either an IEM fellowship or faculty with extensive experience and professional contacts in the RLS may limit creation of similar fellowships in other contexts. At our institution, the PURLS fellowship is integrated alongside pre-existing IEM and EUS fellowships, and faculty from the IEM and EUS fellowships participate in PURLS fellow education according to their expertise. The authors with dedicated PURLS training (SLB and DK) focus on the specific applications of POCUS in RLS. Acquisition and maintenance of functioning ultrasound machines is a possible limitation for any institution utilizing POCUS. This is doubly true in the RLS, but many possible solutions exist, including newer “ultra-portable” machines, a variety of NGOs providing refurbished machines for short- or long-term use in RLS, and the increasing availability of ultrasound machines at institutions in the RLS.

## Conclusion

POCUS plays an important role in clinical practice in many settings, but it is increasingly relevant in the RLS. There exists a significant lack of qualified educators to adequately train new providers in the unique applications of POCUS in RLS. The PURLS curriculum provides a model for producing clinician educators with context-specific expertise in tropical infectious disease, clinical practice in RLS, and ultrasound education who can practice effectively and can train other RLS clinicians in POCUS. We believe this novel fellowship can produce clinicians and educators with the knowledge and ability to help improve POCUS education and, ultimately, patient care, where it is needed most.

## Data Availability

Not applicable.

## Abbreviations

PURLS: Point-of-care ultrasound in resource-limited settings
DTM&H: Diploma of Tropical Medicine and Hygiene
ED: Emergency Department
EUS: Emergency ultrasound
FASH: Focused assessment with sonography in HIV-associated TB
HIV: Human Immunodeficiency Virus
IEM: International Emergency Medicine
POCUS: Point-of-care ultrasonography
QA: Quality assurance
RLS: Resource-limited setting
USA: United States of America
